# Millet Disease

**Published:** 1893-11

**Authors:** T. D. Hinebauch


### MILLET DISEASE.

By T. D. Hinebauch, M. S., V. S.

During the winter of '91 and '92, a disease existed among horses that had been fed on millet. It was known as millet disease, and existed to a great extent wherever millet was used as food. There was an average death rate of from seven to ten per cent. On many farms the death rate was considerably higher, while on others no animal succumbed to the disease. A few of the animals affected remained permanently diseased, the disease having settled in the joints.

The condition of the millet at the time of harvesting seemed to make no difference in regard to the virulence of the attack. That which was cut when about one-fourth headed produced the same results as that which was fully headed, or that which had partly ripened.

The symptoms as observed were as follows: For a number of days previous to the attack, the kidneys acted very freely. This continued for several days, when their action was much less than the normal. Muscles of shoulders, chest, loins and haunches stiff and sore. Later on there is soreness of the joints, usually the stifle and hock. This often changes from one leg to another, or from the hind to the fore extremities. There are well marked symptoms of pain with a slight amount of fever. The temperature usually varies from 102 to 104 degrees. In exceptional instances it may reach 106 degrees, but soon recedes. The pulse is more frequent and hard. The fever is remittent rather than continued and varies according to the intensity of the pain. At no time is the animal free from pain or fever during the course of the disease. It is a continued succession of ups and downs, gradually growing less marked until the disease finally comes to an end. The membranes of the eye redden, tongue coated, mouth hot, dry and sticky, having a peculiar sour odor. Bowels constipated, urine scanty, thick and stringy. The pain in the muscles causes the animal to assume a cramped or drawn together position, with back arched and with a well defined line along the lower end of the ribs. He has no disposition to move, but if made to do so, has a straddling, ungainly, painful gait, frequently groaning at every step. Occasionally the animal lies down and is unable to rise, more from the severe pain than from any changed condition of the muscles or joints. At first there is profuse sweating, especially in the region of the affected muscles; the animal will flinch and show more or less evidence of pain. Or, if pressure be applied to the affected joints, the same result will be manifest. If the horse be down he will lie comparatively quiet, and occasionally make a feeble effort to rise. The intense pain that this movement causes induces quietude again, and it is seldom that he will rise, even if persistently urged to do so. There is loss of appetite, the animal generally assuming a painful expression. Should any other region than the one just indicated be affected, similar symptoms will manifest themselves by special movement or manipulation of the diseased part. When the disease occurs in mares there is frequently slight tumefaction of the vulva, which extends into the vagina a variable distance. Underneath the external membrane the connective tissue is engorged with serum, until in some instances the swelling assumes immense volume. Fomenting the swollen parts for a considerable length of time (from one-half to two hours) reduces them to nearly their normal condition. The effect, however, is not permanent, the parts becoming infiltrated in the course of two or three hours. The above condition exists regardless of pregnancy. Animals not in foal showing the same condition as those which are in foal. I am at a loss to state what should induce the above symptoms unless it be merely external manifestation of a general diseased condition of the generative and urinary organs. Pregnant mares showing the above symptoms do not always abort, but give birth to a healthy foal at the end of the usual period of gestation.

A number of those cases which came under my observation showed well marked pleuritis and pleurodynia, the pleurisy being very severe and accompanied by a fever raging from 103 to 104 degrees. With vigorous treatment it usually subsided within twenty-four to forty-eight hours, the joints then gradually becoming affected until the typical form becomes established.

### DURATION OF THE ATTACK.

There is no definite limit to the time a horse may suffer from a single attack. Mules, which are also subject to the disease, have a severer form, which lasts much longer. As far as my experience goes, I have never seen a mule recover entirely under from five weeks to as many months, and they are more apt to remain permanently unfit for work.

Some cases recover in a week or ten days, while others linger for several weeks or months. This is especially the case if the acute form gives way to the chronic. Colts yield more readily to treatment than older horses. In fact the older the horse the more severe will be the disease, and the longer the attack.

In describing the various cases to which we shall allude, the history, surroundings and treatment of the animals prior to the attack, symptoms, treatment and termination of the disease will be considered in each individual instance. We will not attempt to describe all cases we have treated, but select the first two and the last one, as we consider them a fair average of the total number.

CASE I.

Bay mare eight years old, weighs about 1,150 to 1,200 pounds. In foal, due in two months. Was found lying on her right side. Health previously good. Had never been sick while in possession of present owner, who purchased horse at five years old. Bred and raised in Iowa.

Surroundings.—Barn low, housed forty head of mules and horses. No special means of ventilation. Ceiling covered with frost, which has been gradually melting and dropping down upon the stock, all of which were more or less wet. Fed on millet in good condition, cut when about one-fifth headed out. Grain consists of bran principally, some receiving one feed of oats per day. During the day the stock ran in a yard, having access to wheat straw.

Symptoms.—Animal was found lying on her right side, rising on haunches like a dog and groaning as though in intense pain, breathing distressed, and number of respirations increased to eighteen per minute, flank and shoulder covered with sweat. Manipulation of hock and stifle joints induced severe pain. Pulse eighty, wiry and full. Temperature, 104 degrees F. The animal was raised by means of slings, and after remained in them half an hour, was able to stand without assistance, but would not move. The lips of the vulva were much swollen, having a semi-transparent appearance. The evacuation of the bowels showed the secretion to be normal. Urine scanty, clear and light colored. The breath had a disagreeable, sour odor.

The case was diagnosed as rheumatism, and the following treatment prescribed: Fomentation of the vulva until swelling disappears. Pure water containing nitrate of potash in quantity sufficient to allow two ounces per day. Hay and bran, dry bedding. Use of slings, and internally:

Sodium Salicylate 2 oz.
Aqua 8 oz.
Give one ounce every six hours.

Saw the animal two days later, when a marked improvement was noticed. Temperature, 102.5 F. Pulse, sixty, not nearly so wiry. Respiration 12 per minute. Appetite good. Water taken in moderate quantities. Changed the treatment by discontinuing the nitrate of potash and giving the following:

Sodium Salicylate 2 oz.
Fl. Ext. Gentian 2 oz.
Aqua 8 oz.
Give one ounce three times per day.

Did not see the mare again, but was informed that she entirely recovered in about ten days, and when due gave birth to a healthy foal.

CASE II.

Sorrel mare, eleven years old, due to foal.

History.—Health good. Had never suffered from any disease. Had raised two colts, both of which were healthy. For several days previous to my visit, the mare showed slight lameness, which gradually increased in severity. Loss of appetite. Seemed to drink more than usual. First indications of lameness in left hind leg. Was then called to prescribe for the mare.

Surroundings.—Barn contained carriage room, one box and three single stalls. Ventilation poor, ceiling covered with frost, which was melting and dropping on stock. Floor so wet that bedding was wet all the time.

Feed.—Millet, hay and oat straw. The millet and oats had been seeded together, and both were headed when cut for hay. Grain ration consisted of two quarts nice clean oats as a feed three times per day.

Symptoms.—At time of consultation there was but a small quantity of urine secreted, light colored and clear. Great lameness in left hind leg. All the joints were affected, the hock, stifle and fetlock being especially sore and tender to the touch. Tucked up appearance of the abdomen. Respiration, 12 per minute. Temperature, 102.5 degress F. The animal would stand up part of the time, disliked to lie down, and when down would not rise unless urged to do so. The third day after the attack she foaled a healthy colt. Through carelessness of the attendant the colt became chilled by being allowed to lie in the water which came with it, and died in thirty-six hours. The mare refused to get up, was then placed in slings, and during her endeavors to stand ruptured the tendon of the flexor pedis perforatus of the right hind foot from its insertion. She was then destroyed.

Treatment.—Consisted of stimulants and salycilate of soda and nitrate of potash. There was an improvement up to the time of foaling, but at that time the symptoms became aggravated.

CASE III.

History.—Bay mare, age unknown, had given birth to a healthy foal three weeks prior to my being called. Foal healthy at present time (7 months old).

Surroundings.—Ventilation imperfect. Banked barn, walls dry. Fed on millet hay two-thirds headed out. Oats, three quarts.

Symptoms.—Found the animal lying down, endeavored to get her up by the use of slings, would not bear any weight on hind legs. Pain upon pressure of any of the joints of hind legs, and also in gluteal region. Temperature, 104 F. Respiration, 20. Pulse, 70. Rigors and distressed appearance.

After thirty-six hours' treatment the attendant attempted to raise the mare and let her drop, killing her almost instantly. During the struggle she ruptured the gastrocnemious externus from its insertion, allowing the summit of the os calcis to protrude through the skin.

POST-MORTEM.

I held a post-mortem on both of the hind legs and found them essentially the same in morbid appearance. I will describe the lesions as they occurred in the left hind leg, those of the right leg being fully as severe. The insertion of the abductor magnus was torn from its attachment, and carried with it particles of bone. The portion of bone which was removed was nearly circular in shape, a little pointed at the upper extremity, and in all covered about one and one-half square inches. The region of the popliteus was torn loose, and also carried with it small particles of bone; the bone exposed was somewhat softened, it was also severed from its attachment to the capsular ligament of the stifle joint. The capsular ligament of the patella showed marked infiltrations with a diminished supply of synovial fluid. In fact the absence of synovial fluid was well marked in all the joints, there being scarcely a trace left in some of them. The insertion of the triceps abductor femoris, the tensor fascia lata and quadriceps cruralis were also torn loose from the bone, and as was the case with the other muscles, carried with them particles of bone. The bursa at the side of the hock through which the tendon of the peroneus muscle passed, was dry and contained scarcely a trace of synovial fluid. The parts adhered to each other showing that the condition had existed for some little time previous to death. The gastrocnemius externus was also ruptured from its attachment, allowing the hock to become flexed. The summit of the os calcis protruded through the soft tissues and skin so that it was exposed to sight. No synovial fluid found in the hock joint. The external straight ligament of the patella, the external middle interior sesamoidean ligament, the right branch of the superior sesamoidean, the lateral ligaments of the pastern joint and the two posterior ligaments of the same joint were torn from their attachments. The capsular ligament of the fetlock joint was perforated at its postero inferior aspect, and also near the center of the postero internal aspect.

The joints.—The distal extremity of the femur presented indentations on both condyles and trochlea. On the condyles they were small, pit-like, some round, some oblong, while some were long and narrow, having the appearance of a line. The trochlea was smooth, and at its inferior portion the cartilage had become nearly worn away so that distinct grooves were noticeable where it came in contact with the patella.

The Patella.—The texture of the patella was spongy, being not nearly so hard and compact as the average A portion of its ligamentous attachments had given away, exposing the cancellated structure of the bone. Its posterior surface which articulates with the trochlea of the femur, especially the innermost concavity contained numerous indentations, exhibiting a partial destruction of the cartilage. These indentations were nearly all oval, a few of them triangular in shape, pit like, and some of them quite deep. In the crest inside the lateral borders abrupt eminences composed wholly of cartilage existed.

The Tibia.—The proximal extremity showed indentations similar in character, but not nearly so numerous as those which existed on the distal extremity of the tibia, and also showed more marked deterioration than any of the other joints of the limb. The cartilages in the particular grooves had completely disappeared in the center, leaving the bone exposed. The disease had not existed long enough, evidently to allow porcellaneous deposit to take place; it was somewhat roughened in character. The ridges of the astragalus contained numerous small indentations at their acute angle, and showed an almost entire absence of the articular cartilage.

Fetlock Joint.—Changes similar to those of the femoro patellæ and tibial joint. The pastern joint showed very little change and that on the distal extremity of the os suffraginis. All the particular surfaces of the os corona, of the navicular and os pedis were normal with the exception of two small spots which existed on the articular surface of the os pedis where it articulates with the os corona.

The ill effect of the feeding of millet occurs mostly durıng cold weather, and in horses that are not at work. Whether cold of itself is an important factor, or not, we are unable to say, but we think it has more or less to do with the diseased condition. One very important factor is the want of proper ventilation. The greatest number of affected animals belonging to any single farm were invariably found in barns where ventilation was the poorest. In fact we can only call to mind a very few cases where the ventilation was good. Those cases were less severe than those where the stables were illy ventilated. They also recover much more quickly.

The question of proper ventilation is not so serious a one where extremely low temperatures do not occur. But where there is extreme cold as in our region, the thermometer going as low as 40 to 50 degrees F., and remaining below 20 degrees F. for several weeks at a time, and accompanied by high winds, then the matter of properly ventilating a barn becomes a serious question.

September 7th, 1893, we began a series of experiments to determine accurately the effects of millet upon horses.

We selected three geldings, one three years old, one five years, and the other six years old. The three year old could not be handled satisfactorily, and was thrown out after having been an experiment for five days. The other two were continued over a period lasting from September 7th to October 4th, inclusive. The first period lasted from September 7th to September 20th, it being a preliminary one. During that time the horses received hay and oats. On September 20th the feed was changed from hay to millet, the same number of pounds of millet being given as they had consumed of hay. The millet was less than half headed out, and was secured in fairly good order; the oats were old and in good first class condition; the same oats being fed during both periods. On October 1st, hay was again substituted for millet, in order to place the animals again in a normal condition. The results of this experiment I have tabulated as follows:

| 1893 | | Harry, S. G., 7.9 old. Temp. Fahr. Air. | Horse. | Water. | Hay. | Oats. | Salt. | Urine. | Weight. | Jim, Bl. G., 5.9 old. Temp. Fahr. Air. | Horse. | Water. | Hay. | Oats. | Salt. | Urine. | Weight. |
|---|---|---|---|---|---|---|---|---|---|---|---|---|---|---|---|---|---|
| | | | | lb. | lb. | lb. | | | | | | | | | | | |
| | a.m. | 54 | ... | 30 | 8 | 4 | .. | .... | .... | .. | .... | 15 | 8 | 4 | .. | .... | .... |
| Sept. 7 | n. | 64 | .... | 41 | 8 | 4 | .. | .... | .... | .. | .... | 13 | 8 | 4 | .. | .... | .... |
| | p.m. | 67 | .... | 22 | 8 | 4 | .. | .... | .... | .. | .... | 40½ | 8 | 4 | .. | ... | .... |
| | a.m. | 65 | .... | 20 | 8 | 4 | .. | .... | .... | .. | 99.8 | 35 | 8 | 4 | .. | .... | .... |
| Sept. 8 | n. | 82 | 100 6 | 9 | 8 | 8 | .. | .... | .... | .. | 99.8 | .... | 8 | 4 | .. | .... | .... |
| | p.m. | 83 | 100.2 | 44 | 10 | 4 | . | ... | .... | .. | 100.4 | 41 | 8 | 4 | .. | .... | .... |
| | a.m. | 65 | 100.2 | 29 | 10 | 4 | .. | ... | .... | .. | 99.8 | 36 | 8 | 4 | .. | .... | .... |
| Sept. 9 | n. | 84 | 100.4 | 21 | 10 | 4 | .. | .... | .... | .. | 100. | 11 | 1 | 4 | .. | .... | .... |
| | p.m | 80 | 100.6 | 13 | 14 | 4 | .. | .... | .... | .. | 100.6 | 43.5 | 8 | 4 | .. | .... | .... |
| | | | | | | | | | | | | | | | oz. | | |
| | a m. | 65 | 99.8 | 40.5 | 9 | 4 | 3 | .... | .... | .. | 99.4 | 22 | 7 | 4 | 1 | .... | .... |
| Sept. 10 | n. | 83 | 100.8 | .... | 9 | 4 | .. | .... | .... | .. | 100.2 | 11 | 7 | 4 | .. | .... | .... |
| | p.m. | 75 | 100.4 | 62 | 9 | 4 | .. | .... | .... | .. | 100. | 41 | 7 | 4 | .. | .... | .... |
| | a.m. | 75 | 100.4 | 56 | 9 | 4 | 1 | .... | ... | .. | 100. | 37 | 7 | 4 | 1 | .... | .... |
| Sept. 11 | n. | 89 | 100.2 | 28.5 | 9 | 4 | .. | .... | 1390 | .. | 99.8 | 14.6 | 7 | 4 | .. | .... | 1258 |
| | p.m. | 80 | 100.8 | 55.5 | 9 | 4 | .. | .... | | .. | 101. | 53 | 7 | 4 | .. | .... | |
| | a.m. | 67 | 100. | 15.5 | 9 | 4 | .. | .... | | .. | 100.2 | 11 | 7 | 4 | .. | .... | |
| Sept. 12 | n. | 84 | 100.8 | 14 | 9 | 4 | .. | .... | 1368 | .. | 100.8 | 28 | 7 | 4 | .. | .... | 1238 |
| | p.m. | 84 | 101.6 | 60.5 | 9 | 4 | . | .... | | .. | 101.4 | 37.5 | .. | .. | .. | .. | |
| | | | | | | | | lb. oz. | | | | | | | | lb. oz. | |
| | a.m. | 70 | 101.4 | 35.5 | 9 | 4 | 1 | 17 14 | | .. | 101.2 | 38.5 | 3.5 | 4 | 1 | 12 8 | |
| Sept. 13 | n. | 82 | 101. | 2.5 | 9 | 4 | .. | | 1370 | .. | 100.8 | 13 | 7 | 4 | .. | | 1245 |
| | p.m. | 67 | 100.6 | 47 | 9 | 4 | .. | | | .. | 101. | 27.5 | 7 | 4 | .. | | |
| | a.m. | 54 | 100.4 | 10.5 | 9 | 4 | .. | 17 14 | | .. | 100.6 | 12 | 7 | 4 | .. | 16 8 | |
| Sept 14 | n. | 77 | 100.8 | 23 | 9 | 4 | .. | | 1360 | .. | .... | 14 5 | 7 | 4 | .. | | 1250 |
| | p.m. | 66 | .... | 30 | 9 | 4 | .. | | | .. | .... | 44 | 7 | 4 | .. | | |
| | a.m. | 54 | .... | 20 | 9 | 4 | .. | .... | | .. | ... | 22.5 | 7 | 4 | .. | .... | |
| Sept. 15 | n. | 62 | 100.2 | 11.5 | 9 | 4 | .. | .... | 1340 | .. | 100. | 18.5 | 7 | 4 | .. | .... | 1235 |
| | p.m. | 58 | 100.4 | 36.5 | 9 | 4 | 1 | .... | | .. | 100.6 | 25 | 7 | 4 | 1 | .... | |
| | a.m. | 41 | 100.4 | 21 | 9 | 4 | .. | .... | | .. | 100. | 15.5 | 7 | 4 | .. | .. . | |
| Sept. 16 | n. | 62 | 101.2 | 22.5 | 9 | 4 | .. | .... | 1350 | .. | 101. | 26 | 7 | 4 | . | .... | 1245 |
| | p.m. | 62 | 100.6 | 37.5 | 9 | 4 | 1 | .... | | .. | 100.6 | 26.5 | 7 | 4 | 1 | ... | |
| | a.m. | 56 | 100.2 | 3 | 9 | 4 | .. | 13 8 | .... | .. | 100.2 | 6 | 7 | 4 | .. | 13 3 | .... |
| Sept. 17 | n. | 76 | 100.4 | 45.5 | 9 | 4 | .. | | .... | .. | 99.8 | 45.5 | 7 | 4 | .. | | .... |
| | p.m. | 74 | 100.6 | 36 | 9 | 4 | 1 | | .... | .. | 101.2 | 28 | 7 | 4 | 1 | | .... |
| | a.m. | 48 | 100.8 | 40 | 9 | 4 | .. | .... | | .. | 100.2 | 28.5 | 7 | 4 | .. | 15 3 | |
| Sept. 18 | n. | 59 | .... | .... | 9 | 4 | .. | .... | 1340 | .. | ... | 9 | 7 | 4 | .. | spg. | 1225 |
| | p.m. | 53 | 100. | 38 | 9 | 4 | 1 | .... | | .. | 99.8 | 27½ | 7 | 4 | 1 | 1.097 | |
| | | | | | | | | lb. oz. | | | | | | | | lb. oz. | |
| | a.m. | 40 | 100.8 | 27 | 9 | 4 | .. | 15 14 | | .. | 101. | 26 | 7 | 4 | .. | 10 11 | |
| Sept. 19 | n. | 56 | 100.2 | 3 | 9 | 4 | .. | | 1335 | .. | 100. | .... | 7 | 4 | .. | | 1230 |
| | p.m. | 54 | 100.4 | 10 | 9 | 4 | 1 | | | .. | 100.4 | 39½ | 7 | 4 | 1 | | |
| | a. m. | 57 | 100.2 | 19½ | 9 | 4 | .. | 14 0 | | .. | 100.4 | 20 | 7 | 4 | .. | 10 8½ | |
| Sept. 20 | n. | 70 | 101. | .... | 9 | 4 | .. | | 1340 | .. | 101. | .... | 7 | 4 | .. | | 1230 |
| | p.m. | 75 | 100 2 | 56 | 9 | 4 | 1 | | | .. | 100. | 53 | 7 | 4 | 1 | | |
| | a.m. | 51 | 100.2 | 44 | 9 | 4 | .. | 21 0 | | .. | 101. | 35 | 7 | 4 | .. | 18 8 | |
| Sept. 21 | n. | 58 | 100.8 | 23.5 | 11 | 4 | .. | spg. | 1338 | .. | 100.4 | 23.5 | 9 | 4 | .. | spg. | 1215 |
| | p.m. | 56 | 100.6 | 53.5 | 9 | 4 | 1 | 1.046 | | .. | 100.4 | 34.5 | 7 | 4 | 1 | 1.044 | |
| | a. m. | 41 | 100.6 | .... | 9 | 4 | .. | .... | | .. | 100.8 | .... | 7 | 4 | .. | 23 1½ | |
| Sept. 22 | n. | 57 | 99.8 | 39.5 | 9 | 4 | .. | ... | 1340 | .. | 98.8 | 41.5 | 7 | 4 | .. | spg. | 1238 |
| | p.m. | 53 | 100.4 | 45 | 9 | 4 | 1 | .... | | .. | 100.4 | 85 | 7 | 4 | 1 | 1.045 | |

Temperatures are Fahrenheit. Weights are lb. and oz. Urine record extends from 6 P.M. to 6 P.M. (24 hrs.)

At 6 P. M. of the 20th, millet was substituted for hay, and continued as the coarse feed. The millet was nearly one-half kneaded out. No heads were fully developed.

| 1893. | | Harry, S. G., 7.9 old. Temp. Fahr. Air. | Horse. | Water. | Millet. | Oats. | Salt. | Urine. | Weight. | Jim, Bl. G., 5.9 old. Temp. Fahr. Air. | Horse. | Water. | Hay. | Oats. | Salt. | Urine. | Weight. |
|---|---|---|---|---|---|---|---|---|---|---|---|---|---|---|---|---|---|
| | | | | | | | | lb. oz. | | | | | | | | lb. oz. | |
| | a.m. | 36 | 100. | .... | 9 | 4 | 1 | 33 11½ | | 36 | 99.6 | .... | 7 | 4 | 1 | 27 5 | |
| Sept. 23 | n. | 50 | 99.2 | 35 | 9 | 4 | .. | spg. | 1342 | 50 | 100. | 42½ | 7 | 4 | .. | spg. | 1212 |
| | p.m. | 49 | 100. | 50 | 9 | 4 | .. | 1.038 | | 49 | 100.6 | 13½ | 7 | 4 | .. | 1.038 | |
| | | | | | | | | lb. oz. | | | | | | | | lb. oz. | |
| | a.m. | 37 | 101. | 29 | 9 | 4 | 1 | 33 11½ | .... | 37 | 100.4 | 36 | 7 | 4 | 1 | 28 8½ | |
| Sept. 24 | n. | 47 | 99.8 | 8 | 9 | 4 | .. | spg. | .... | 47 | 98.8 | .... | 7 | 4 | .. | spg. | .... |
| | p.m. | 42 | 99.4 | 39½ | 9 | 4 | .. | 1.031 | .... | 42 | 100. | 35½ | 7 | 4 | .. | 1.032 | .... |
| | | | | | | | | lb. oz | | | | | | | | lb. oz. | |
| | a.m. | 32 | 100.6 | 33 | 9 | 4 | 1 | 26 11 | | 32 | 100.4 | 32½ | 7 | 4 | 1 | 24 12 | |
| Sept. 25 | n | 48 | 99.8 | 43 | 9 | 4 | .. | spg. | 1355 | 48 | 99.6 | 26 | 7 | 4 | .. | spg. | 1225 |
| | p.m. | 42 | 99.4 | 37 | 9 | 4 | .. | .... | | 42 | 100.4 | 34 | 7 | 4 | .. | .... | |
| | | | | | | | | lb. oz. | | | | | | | | lb. oz. | |
| | a.m. | 35 | 101. | .... | 10 | 4 | 1 | 34 3 | | 35 | 106.6 | 26½ | 8 | 4 | 1 | 29 1 | |
| Sept. 26 | n. | 48 | 100.6 | 45½ | 10 | 4 | .. | spg. | 1350 | 48 | 100.8 | 38 | 8 | 4 | .. | spg. | 1235 |
| | p.m. | 46 | 100.6 | 33½ | 10 | 4 | .. | 1.041 | | 46 | 100.4 | 34½ | 9 | 4 | .. | 1.037 | |
| | | | | | | | | lb. oz. | | | | | | | | lb. oz. | |
| | a.m. | 30 | 101. | 34½ | 10 | 4 | 1 | 34 2 | | 35 | 100.4 | 28 | 9 | 4 | 1 | 35 8 | |
| Sept. 27 | n. | 50 | 100. | 31 | 10 | 4 | .. | spg. | 1350 | 50 | 99.8 | 20 | 9 | 4 | .. | spg. | 1238 |
| | p.m. | 48 | 100.4 | 52½ | 10 | 4 | .. | 1.C41 | | 48 | 100 6 | 34 | .. | 4 | .. | 1.032 | |
| | | | | | | | | lb. oz. | | | | | | | | lb. oz. | |
| | a.m. | 39 | 100.4 | 12 | 10 | 4 | 1 | 36 5½ | | 39 | 100.6 | 19½ | 8 | 4 | 1 | 26 8 | |
| Sept. 28 | n. | 58 | 100.4 | 34½ | 10 | 4 | .. | spg. | 1358 | 58 | 100. | 27 | 6 | 4 | .. | spg. | 1235 |
| | p.m. | 57 | 101. | 56½ | 10 | 4 | .. | 1.035 | | 57 | 100.4 | 35 | 7 | 4 | .. | 1.032 | |
| | | | | | | | | lb. oz. | | | | | | | | lb. oz. | |
| | a.m. | 53 | 100.6 | .... | 10 | 4 | 1 | 34 3 | | 53 | 100.8 | 28 | 7 | 4 | 1 | 29 1 | |
| Sept. 29 | n. | 51 | 100.2 | 40½ | 10 | 4 | .. | spg. | 1360 | 51 | 100.4 | 18 | 7 | 4 | .. | spg. | 1250 |
| | p.m. | 49 | 101. | 42½ | 10 | 4 | .. | 1.041 | | 49 | 100.2 | 35½ | 7 | 4 | .. | 1.037.5 | |
| | | | | | | | | lb. oz. | | | | | | | | lb. oz. | |
| | a m. | 48 | 100.4 | .... | 10 | 4 | .. | 35 8 | | 48 | 100.4 | 11 | 7 | 4 | .. | 29 2 | |
| Sept. 30 | n. | 52 | 101. | 35 | 10 | 4 | .. | spg. | 1362 | 52 | 100.4 | 25 | 7 | 4 | .. | spg. | 1258 |
| | p.m. | 41 | 101.8 | 69 | 10 | 4 | .. | 1.043 | | 41 | 100.6 | 44½ | 7 | 4 | .. | 1.041 | |
| | | | | | Hay. | | | | | | | | | | | | |
| | a.m. | 51 | 100.6 | 6 | 9 | 4 | 1 | .... | .... | .. | 98.8 | 14½ | 7 | 4 | 1 | .... | .... |
| Oct. 1 | n. | 51 | 100.8 | 27 | 9 | 4 | .. | .... | .... | .. | 100. | ... | 7 | 4 | .. | .... | .... |
| | p.m. | 47 | 101. | 47 | 9 | 4 | .. | .... | .... | .. | 100.4 | 36 | 7 | 4 | .. | .... | .... |
| | | | | | | | | lb. oz. | | | | | | | | lb. oz. | |
| | a.m. | 37 | 100.4 | .... | 9 | 4 | 1 | 29 5 | | .. | 100.2 | 23 | 7 | 4 | 1 | 24 3 | |
| Oct. 2 | n. | 39 | 100.8 | 14 | 9 | 4 | .. | spg. | 1362 | .. | 100.4 | .... | 7 | 4 | .. | spg. | 1252 |
| | p.m. | 43 | 100.4 | 28 | 9 | 4 | .. | 1.035 | | .. | 101. | 45½ | 7 | 4 | .. | 1.043 | |
| | | | | | | | | lb. oz. | | | | | | | | lb. oz. | |
| | a m. | 40 | 100.4 | 25 | 9 | 4 | 1 | 15 11 | | .. | 101. | 25 | 7 | 4 | 1 | 10 4½ | |
| Oct. 3 | n. | 57 | 100.4 | 2 | 9 | 4 | .. | spg. | 1364 | .. | 101. | 10 | 7 | 4 | .. | spg. | 1252 |
| | p m. | 52 | 100.6 | 52½ | 9 | 4 | .. | 1.049 | | .. | 100.6 | 42 | 7 | 4 | .. | 1.051 | |
| | | | | | | | | lb. oz. | | | | | | | | lb. oz. | |
| | a.m. | 44 | 101. | 32 | 9 | 4 | 1 | 17 14 | | .. | 100.6 | 15 | 7 | 4 | 1 | 14 10 | |
| Oct. 4 | n. | 54 | 100.2 | ... | 9 | 4 | .. | spg. | 1358 | .. | 100 4 | 9½ | 7 | 4 | .. | spg. | 1245 |
| | p.m. | 52 | 100.4 | 49 | 9 | 4 | .. | 1.041 | | .. | 100.6 | 47 | 7 | 4 | .. | 1.045 | |

NOTE—Both horses were changed from millet to hay the evening of October 1st. On the 27th of September Harry passed urine twenty-seven times; exhibited considerable pain. Jim had two attacks of colic September 22 and September 27. From the 23d to the 27th Jim moved about with a stradling gait when first moved. During the whole trial both horses were either driven or ridden for exercise. The distance was from eight to twelve miles daily.

During the winter we shall continue our experiments in that line, using millet which is all headed for one lot, another lot partially matured, and a third lot ready for seed. We shall also feed seed as a grain feed instead of oats. Another line of experiment will be for the purpose of determining whether poorly gathered millet produces a more aggravated form of the disease than when it is gathered in good condition. We have found that millet is a very hard crop to secure in excellent condition. It readily moulds if there is much moisture during the process of curing. Should a rain set in at that time, and continue for a day or two, the millet invariably moulds to a greater or less extent. In fact it is practically impossible to secure it so that the dust does not rise from it when it is shaken.

The chemical analysis was kindly made for me by Prof. Ladd and his assistant, Prof. Whalen. They were conducted with a view of only determining the amount of nitrogen in each sample, and the amount of dry matter.

*To be Continued.*

